# Lingual Abscess: Predisposing Factors, Pathophysiology, Clinical Manifestations, Diagnosis, and Management

**DOI:** 10.1155/2018/4504270

**Published:** 2018-11-07

**Authors:** Chonticha Srivanitchapoom, Kedsaraporn Yata

**Affiliations:** Otolaryngology Unit, Phayao Hospital, Phayao, Thailand

## Abstract

Lingual abscess is a rare disorder, and current knowledge regarding clinical manifestations and treatment modalities has not been well established. This study presented 6 cases of lingual abscess patients between January 2012 and December 2017. There were three men and three women. Median age was 54 years. Odynophagia and local pain were the common presenting symptoms. Local trauma was the main predisposing factor of anterior abscess, while lingual tonsillitis or infected thyroglossal cyst was the predisposing factor of posterior abscess. An impending airway obstruction was identified in two patients, requiring tracheostomy. All patients achieved an excellent outcome with a combination of surgical drainage and proper antibiotics as well as using proper investigation for detecting unusual areas of lingual abscess. According to the data from the study's results and review of the relevant literature, an abscess located at the anterior two-thirds of the tongue is easy to diagnose while the posterior one-third of the tongue abscess is relative difficulty. Using contrast-enhanced computed tomography increases diagnostic accuracy, especially on the tongue base and deep space infection. The management strategies include (1) protecting the airway, (2) draining the abscess by needle aspiration or surgery, and (3) administering antibiotics early. Our series showed a superiority of surgical drainage when the patients present with marked tissue edema, deep loculated infection, and airway obstruction.

## 1. Introduction

Lingual abscess is an infectious process within the tongue parenchyma [1, 2]. Clinical characteristics, predisposing factors, and treatment options of lingual abscess have not been well organized in the literature [3–5]. Most of the knowledge has been provided at the case report level [6–11]. Most data were reported in the pre-antibiotics era which showed a mortality rate of 3%; additionally, disease-related death was usually complicated by asphyxia due to upper airway obstruction [1, 3–5, 8, 9, 11–16]. In the modern treatment era, early detection, advanced investigations, and effective management should reduce the morbidity and mortality rate [9, 10, 14, 16]; however, no data currently address the clinical outcome related to morbidity and mortality of patients with lingual abscess.

The current study presents a series of six patients with lingual abscess who had a different degree of severity in clinical presentations with review and summarizes the previous literature relevant to our patients' conditions in terms of possible pathophysiology, pathogens, predisposing factors, clinical manifestations, diagnosis, and proper management.

## 2. Materials and Methods

The retrospective medical chart review of patients with lingual abscess, who were admitted between January 2012 and December 2017, was performed at the otolaryngology unit. All demographic data, clinical presentations, abscess location, associated risk factors, investigations, surgical drainage, anaesthetic procedure, organism, and managements were studied. Furthermore, patients with airway obstruction and treatment intervention were also recorded. The inclusion criteria were patients admitted in the inpatient unit to receive abscess drainage and antibiotics administration. Some of the patients who presented with deep neck infection concomitant with tongue abscess were included. The exclusion criteria were patients who had underlying diseases of the tongue. The protocol of the investigation has been approved by the Institutional Review Board.

The relevant literature was identified by searching the following databases on March, 2018: Pubmed, Cochrane database of Clinical Trails, Science Direct, and Google Scholar. Lingual abscess or tongues abscess was used as a keyword. We searched for all types of studies about tongue abscess, and 85 articles were identified; however, 23 studies were excluded during the initial search as they were in non-English language. Five case series and 57 case reports met the criteria. All case series were summarized and compared to the result of this case series. In addition, some case reports which had different clinical course were reviewed by focusing on clinical manifestations, location of the abscess, concomitant deep space infection, presentation of airway obstruction, and management.

## 3. Results

### 3.1. Case Series

#### 3.1.1. Case 1

A 55-year-old Thai man presented with dyspnea for 1 day. The patient had a history of odynophagia and dysphagia for a week. He had a history of poor oral hygiene. On physical examination, he had low-grade fever (38°C), dyspnea, and marked swelling of the base of tongue (BOT) with partial occlusion of the oropharyngeal airway. Complete blood count (CBC) showed his white blood cell (WBC) count was 9300/mm3 with predominant neutrophil. Orobuccal computed tomography (CT) scan revealed the large abscess at the BOT. Tracheostomy and surgical drainage were performed.* Streptococcus viridians* was identified from pus culture. Amoxicillin-clavulanic acid 1.2 g 8 hourly plus ceftriaxone 2 g once daily were administrated for 2 weeks with an excellent outcome. The patient could be decannulated after the sixth day of treatment with airway patency.

#### 3.1.2. Case 2

A 52-year-old Thai man presented with a painful tongue and odynophagia for 7 days. The patient had a history of well-controlled diabetes mellitus (DM). He denied either trauma or infection at orobuccolingual regions. Also, the patient had poor oral hygiene. Physical examination showed the patient was afebrile (36.6°C). The antero-lateral aspect of the tongue was swollen and fluctuation. The airway was patent. CBC showed WBC was 4500/mm3 with neutrophilic predominance. Surgical drainage was performed under local anaesthesia. The pus culture did not show any organisms. Empirical antibiotics with amoxicillin-clavulanic acid 1.2 g 8 hourly and ceftriaxone 2 g once daily were administrated for 10 days, and the outcome was good.

#### 3.1.3. Case 3

A 52-year-old Thai woman reported having odynophagia and dysphagia for 1 week. She took amoxicillin for 5 days without signs of improvement. Her symptoms worsened, as did limitation of her tongue movement. She denied local trauma of orobuccolingual regions. Examination showed that her floor of mouth (FOM) and BOT were swollen without airway obstruction. She was afebrile (36.6°C), but her WBC was 14500/mm3. Contrast-enhanced CT scan demonstrated an abscess at the sublingual space and BOT. The pus was drained with the patient under general anaesthesia. The organism was identified as* Acinetobacter lwoffii*. Clindamycin 600 mg 8 hourly and ceftriaxone 2 g once daily were prescribed for 2 weeks, with good clinical response.

#### 3.1.4. Case 4

**Figure 1:**
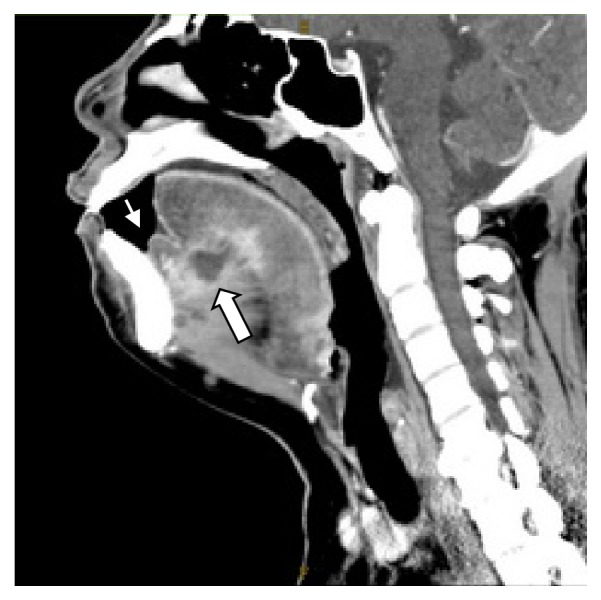
Contrast-enhanced CT scan showed the abscess confined at the ventral aspect of the tongue with sublingual space cellulitis (thick arrow) and marked swelling of anterior floor of mouth was demonstrated (thin arrow).

A 46-year-old Thai woman with poorly controlled DM presented to the emergency department with dyspnea for 1 day. She had odynophagia and dysphagia for 4 days. She denied a history of trauma at orobuccolingual regions. On physical examination, her body temperature was 37.5°C. Limitation of tongue movement and swelling of the tongue and FOM were observed. The oropharyngeal airway was partially obstructed. Contrast-enhanced CT scan showed the abscess confined to the ventral aspect of the tongue with sublingual space cellulitis (Figure 1). CBC showed WBC was 12,100/mm3 with neutrophilic predominance. The patient underwent tracheostomy and surgical drainage under general anaesthesia. The tracheostomy tube was safety removed 5 days after the operation. Beta-haemolytic non-group A, B, D* Streptococcus *Spp. was identified; then, clindamycin 600 mg 8 hourly and ceftriaxone 2 g once daily were administrated intravenously, and all symptoms were completely resolved 2 weeks after initiation of treatment.

#### 3.1.5. Case 5

**Figure 2:**
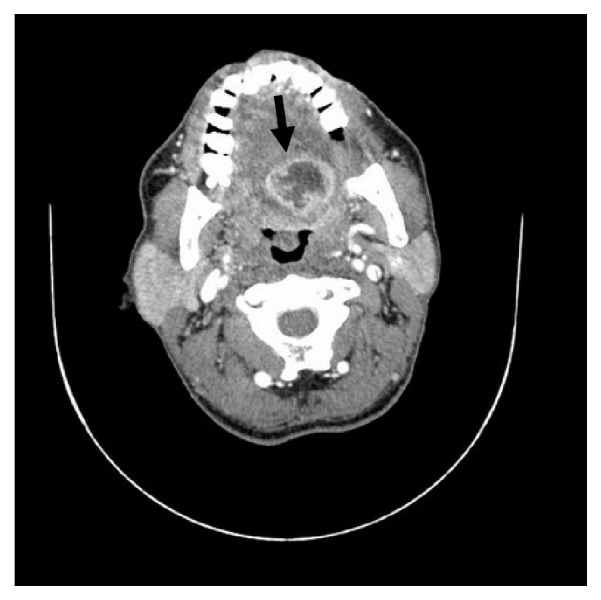
Contrast-enhanced CT scan demonstrated an abscess at left posterior tongue (arrow).

**Figure 3:**
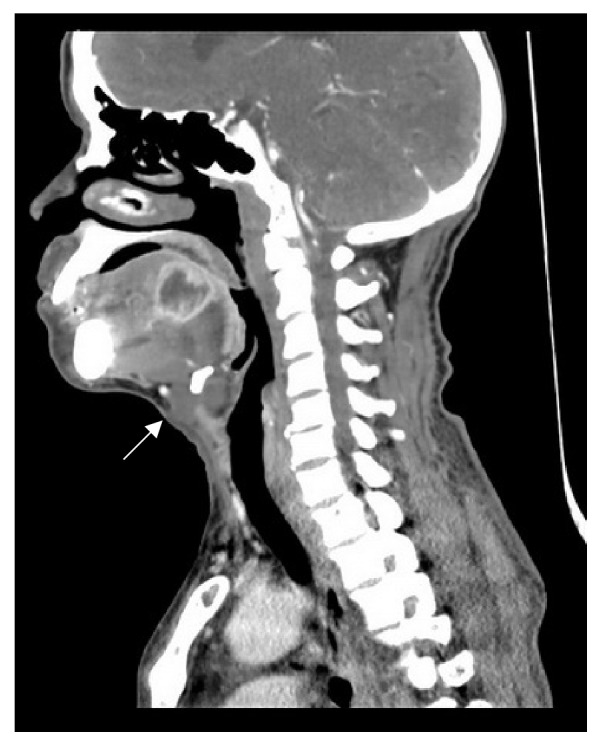
Contrast-enhanced CT scan demonstrated an abscess at left posterior tongue with thyroglossal duct cyst (arrow) was also identified without feature of rim enhancement.

A 58-year-old Thai male was admitted due to pain in the BOT with referred pain to the ear for 1 week. He had poor oral hygiene. He took amoxicillin for 5 days without clinical improvement. On physical examination, he was afebrile (37.3°C). Pain was detected at the left side of BOT just behind the circumvallate papillae with marked fluctuation. The other orobuccal regions were normal without evidence of airway obstruction. CBC showed WBC was 5500/mm3. Contrast-enhanced CT scan demonstrated an abscess at the left posterior tongue (Figure 2). In addition, an incidental thyroglossal duct cyst was identified without sign of infection (Figure 3). The patient underwent surgical drainage under general anaesthesia.* Streptococcus viridans* was identified. Amoxicillin-clavulanic acid 1.2 g 8 hourly and ceftriaxone 2 g once daily were prescribed for 2 weeks, with an excellent outcome.

#### 3.1.6. Case 6

A 59-year-old Thai woman reported having mass within her tongue for 10 days. She had mild degree of pain and took amoxicillin-clavulanic acid for 7 days. Her tongue's mass was still the same size while the pain was resolved. Her oral hygiene was good with no active dental and periodontal conditions. Physical examination showed the patient was afebrile (37°C). The firm mass within the antero-midline of the tongue of about 1.5*∗*1.5 cms in size was palpated without any sign of inflammation. CBC showed WBC was 5800/mm3 with neutrophilic predominance. The patient was informed about the treatment option and she decided to remove the mass under general anaesthesia. Intraoperative finding showed well circumscribed loculated abscess and the pus was drainage. Pathologic report for surrounding tissue was acute and chronic inflammation with abscess formation. The pus culture did not show any organisms. Empirical antibiotic with amoxicillin-clavulanic acid 1.2 g 8 hourly was administrated for 1 week, and the outcome was good.

All patients' demographic data are described in Table 1, and the details of investigation, treatment and clinical outcome are shown in Table 2.

### 3.2. Review of the Relevant Literature

There were 5 case series of 13 patients that correlated with this study (Table 3) [3, 6, 11, 17, 18]. According to the articles, thirteen patients (nine men and four women) were identified, three of which were children. Median age was 41 years. Most of the patients had not showed any diseases comorbidity. The most common presenting symptoms were painful tongue, odynophagia, and tongue swelling. However, Antoniades and colleagues [3] reported two patients with immunocompromised host presenting with serious symptom of airway distress. The predisposing factor was also identified by posttraumatic dental procedure and odontogenic infection. Data confirmed that history taking, physical examination, and needle aspiration can provide the exact diagnosis [3, 6, 11, 17]. In addition, contrast-enhanced CT scan was helpful in diagnosing [6, 18].

The literature review also emphasized that drainage of the pus collection combined with board spectrum antibiotics was the effective management strategy for lingual abscess patients [3, 6, 11, 17, 18]. The method of drainage included needle aspiration and open surgical drainage [3, 6, 11, 17, 18]. However, if concomitant sublingual space infection was identified, incision and drainage of the space with needle aspiration to the tongue were preferred as a choice of treatment [3]. For all abscess locations, the procedure was usually performed under local anaesthesia while general anaesthesia was selected for children [18]. There was no patient who presented with an impending airway obstruction; thus the airway intervention was not well described. The treatment outcome was good for all literature and no disease-related death was reported. Summary of the clinical manifestation, extension of the disease, airway management, and treatment technique of case series from systematic review and this study were presented in Table 4.

## 4. Discussion

### 4.1. Epidemiology and Predisposing Factors

According to the few cases, the incidence of lingual abscess has not been established. Much evidence has concluded this disorder affects males more often than females [1, 9–11]; however, Munoz and colleagues reported no sexual predominance [2]. There was no significant age factor [1]; however, the age range between 25 and 50 years was identified, which is similar to the median age of onset in our series [2, 9, 11]. However, the age of onset of lingual abscess could be as low as 7 weeks, as reported by Sander and colleagues [8]. Kuge and colleagues also reported recurrent abscess in child with 14 months old [19]. Similar to previous evidence, the predisposing factor of our patients was poor oral hygiene. In addition, the predisposing factors reported in the previous literature were heavy smoking and using toothpicks, which were usually found in males [9, 10]. Local trauma, dental carries, penetration of foreign bodies (e.g., fish bones), recent periodontal antibiotic injection, and existing infections secondary to systemic illness such as scarlet fever or influenza were reported as the predisposing factors [1, 2, 5, 9, 11, 16, 20]. In addition, infected follicular lingual tonsils and infected thyroglossal cysts are the most common factors for a posterior lingual abscess [7–9, 12]. Patient number 5 in this series presented with thyroglossal duct cyst simultaneously with the tongue abscess; however, poor oral hygiene was considered a predisposing factor because the abscess was located more anteriorly than is usual on the foramen cecum. An immunocompromised host, such as those with DM and human immunodeficiency virus infection, taking immunosuppressive drugs is at greater risk of infection [3, 4, 12–14]. However, spontaneous abscess development with an unknown etiology was considered [1, 10, 11, 14, 16].

### 4.2. Pathophysiology and Pathogens

Pathophysiology of a lingual abscess may be explained by disruption or dysfunction of protective factors and invasion of a pathologic organism [3, 8]. Usually, protective mechanisms of infection include (1) amount of high vascularity and lymphatic supply, (2) tightness of tongue musculature, (3) integrity of squamous epithelium covering the tongue surface, (4) constant tongue mobility, and (5) continuous turnover of saliva (cleaning, bacteriostatic, lubricant, immunology) [3, 7, 9, 11, 15, 21]. If one of the protective mechanisms is defective, the pathologic organism can pass through to the tissue and infect and damage the underlying tissues before forming an abscess [3, 8]. In addition, obstruction of the mucous gland and lymph follicle at BOT beneath the epithelium can be a cause of abscess formation [7, 10]. The common pathogens which were often reported in previous literature were normal flora of the oral cavity and oropharynx, including* Streptococcus *Spp.,* Staphylococcus *Spp.,* Haemophilus *Spp.,* Bacteroides* Spp.,* Fusobacterium* Spp., and anaerobic bacteria [1, 2, 4, 8, 9, 13, 14, 22]. Similarly to this case series, three from six patients showed* Streptococcus *Spp. from the bacterial culture; however the anaerobic bacterial culture was not performed in this study. In addition, some rare organisms were also reported, such as* Mycobacterium* Spp.,* Actinomyces *Spp., and* Streptococcus miller* [6, 14, 15, 23]. However, partial antibiotic treatment may cause the culture not to grow [12].

### 4.3. Clinical Manifestations and Differential Diagnosis

Many authors subdivided the location of the lingual abscess into oral and BOT [3, 4, 12, 16, 21] following their different clinical manifestations. An anterior lingual abscess is easier to diagnose [2, 21]. The patients usually report odynophagia, dysphagia, painful tongue, limitation of tongue motion, tongue swelling, and speech difficulty and are afebrile [1–3, 6, 10, 11, 13, 15]. On the other hand, BOT abscess presents with referred otalgia, as in case number 5, dysphonia, acute dysphagia, and severe pain on suprahyoid region and during tongue protrusion [3, 4, 6, 10–12, 16, 24]. Harrington and Sander emphasized that posterior lingual abscess increased the potential for upper airway obstruction [8, 14] because of tongue parenchymal edema that obscured an oropharynx level [3, 4, 8], while in our case series an anteroventral tongue abscess concomitant with sublingual space infection also had a potential of airway obstruction. Mortality was high if the patients presented with clinical sepsis, edema of epiglottis, and descending mediastinitis [1, 2, 5, 12, 16, 21]. Differential diagnosis of lingual abscess includes neoplasm, arteriovenous malformation, cystic lesion, haemorrhage, infarction, pseudoaneurysm of lingual artery, ischaemia from giant cell arteritis, angioedema, actinomycosis infection, and acute epiglottitis [2–4, 9, 10, 13, 16, 19, 25, 26], while abscess at the BOT should be differentiated from lingual tonsillar abscess, intralingual thyroglossal duct cyst, and lingual thyroid cyst [6, 7, 26].

According to this study, all patients presented with acute form of the lingual abscess which was similar to most of the previous literature. On the other hand, chronic form of lingual abscess can present as a clod abscess [23]. Vishwakarma and colleagues [23] reported the lingual tuberculosis patient who had nonpainful tongue abscess for 3 months. Tuberculous of the tongue is the most common site of oral cavity tuberculosis which may be primary or secondary with pulmonary tuberculosis. Both immunocompetent and immunodeficiency host can be infected by* Mycobacterium* Spp. [27, 28]. Lateral border of the oral tongue is the most common affected site [23, 27] while the base of tongue was also identified [29]. The lesion is characterized by nonpainful tongue mass, an ulcer, a fissure, tuberculoma, diffuse glossitis, and nonhealing tongue lesion [23, 27, 29]. The diagnosis based on tissue biopsy shows epitheloid cell granulomas with Langhans type of giant cells [27]. Microscopy and culture of pus demonstrated Acid Fast Bacilli [23, 27].

### 4.4. Diagnosis

Careful history taking and physical examination, including orolingual palpation, help provide the exact diagnosis [8, 9]. Laboratory testing, including needle aspiration and imaging, also provides further information. Most of the patients usually present with normal levels of WBC or slight leucocytosis [5, 9, 11], similar to the patients in our series. Needle aspiration confirms a diagnosis and also treatment especially when an abscess occurs in the anterior part of the tongue [3, 16]. Contrast-enhanced CT scan helps diagnose a posterior lingual abscess and cellulitis and provides the differential diagnosis of other possible lingual masses [4–6, 9, 10, 12, 13, 16] as well as helping clinicians evaluate the concomitant deep neck infection. Another advantage of CT scans is providing information of the extralingual structure, such as the thyroid gland [9]. In this case series, CT scan was performed to evaluate the disease extension before drainage. However if the disease can be clearly identified and concomitant deep neck infection was not suspected, as in case 2 and case 6, the imaging can be skipped. Magnetic resonance imaging (MRI) is another valuable tool for evaluating soft-tissue structures and abscesses [2, 4]. However, a disadvantage of MRI is that it is time consuming, which is unsuitable for patients with impending respiratory failure due to airway compromise. Osammor et al. and Kulkarni et al. described the advantages of ultrasonography (US) as being a nonionized method, noninvasive, comfortable, and cost-effectiveness [26, 30]. Hypoechoic lesion surrounded by hyperechoic ring is the typical finding of an abscess on US [2]. Furthermore, US can differentiate a cyst, lingual arterial aneurysm, and abscess [5, 9]. However, the limitations of this technique are as follows: (1) the result of this technique depends on the operator's experience and (2) this technique must be performed intraorally, which is truly uncomfortable due to pain, and it is hard to assess the deeper lesions such as the posterior part of the tongue [26].

### 4.5. Management

The keys to success in managing lingual abscess comprise three domains, including (1) maintaining airway, (2) draining the abscess, and (3) administering antibiotics [1, 4, 9, 10, 21], which were applied in all our cases. Maintaining the airway is truly important, especially for posterior third lingual abscesses or abscesses located at BOT [3, 12]. However, if an abscess forms in the anterior part of the tongue but is concomitant with sublingual space infection, as in case number 4, the risk of impending airway obstruction is also high. The mechanism of airway obstruction is similar to that in Ludwig's angina. The physician should closely observe the clinical symptoms and promptly perform tracheostomy if indicated [3, 12]. Draining the abscess was described in two methods: needle aspiration and open surgical drainage [4, 6, 9, 10, 12]. Both techniques can be performed via a dorsal or sublingual approach [12], while surgical drainage is preferred in a shallow incision at BOT or FOM to avoid injury to lingual artery [7]. The advantage of needle aspiration is avoiding general anaesthesia and exacerbation of the airway oedema by endotracheal intubation; in addition, repeated aspiration can be considered [6, 12]. This method was encouraged when the abscess was located at the anterior tongue and some selected cases when the abscess appeared at BOT [2–4, 6]. On the other hand, surgical drainage can have more advantages, including a large amount of pus drainage, collection of tissue for biopsy if malignancy is suspected [9], prevention of reinfection due to open drainage [14], and dressing. Therefore, in our series, the authors were encouraged to perform open surgical drainage to achieve a good outcome.

Empirical antibiotics should be broad spectrum to cover the possible organisms originating from the oral cavity and oropharynx [9, 12, 13] which are usually comprise gram-positive, anaerobic, and gram-negative bacteria. Amoxicillin-clavulanic acid [9], clindamycin [9, 14], or a combined agent such as amoxicillin-clavulanic acid or clindamycin or metronidazole plus cephalosporin groups [3–6, 12] may be proper regimens in this situation. However, antibiotic treatment should be adjusted according to the culture and sensitivity result of the pathogen [9, 10]. No consensus for duration of antibiotic therapy [4, 13] has been proposed. Sánchez Barrueco and colleagues suggested administering antibiotics for at least 1 week [12], while Vellin and colleagues preferred giving antibiotics longer (2-3 weeks) [4]. In our case series, 2-week administration of antibiotics was preferred for eradicating infection in moderate to severe cases. Some authors preferred a corticosteroid to decrease the inflammatory tissue oedema, especially after needle aspiration [4, 12, 21]. All patients in our series showed clinical improvement within 3-5 days, which was similar to the results of Bernadini [1].

## 5. Conclusion

Clinical spectrum of lingual abscess can range from mild tongue pain to severe upper airway obstruction. Local trauma is the most common predisposing factor of anterior lingual abscess, while lingual tonsillitis or infected thyroglossal cyst are the main predisposing factors of infection of the tongue base. Diagnosis of an anterior lingual abscess is much easier than diagnosis in the posterior part. Contrast-enhanced CT helps diagnose a posterior lingual abscess. To minimize the morbidity and mortality rate, management strategies include (1) maintaining the airway, (2) draining the abscess by using needle aspiration or surgical drainage, and (3) administering antibiotics early, which is guided by bacterial culture. The authors suggested open surgical drainage when the patients present with marked tissue edema, deep space infection, and airway compromise. Conclusions are supported by the provided data. Treatment outcome was usually good. The mortality rates are less than 3% in the modern antibiotic era.

## Figures and Tables

**Table 1:** Demographic data.

	Patient 1	Patient 2	Patient 3	Patient 4	Patient 5	Patient 6
Sex	Man	Man	Woman	Woman	Man	Woman

Age (year)	55	52	52	46	58	59

Underlying diseases	None	DM & HT	None	DM & HT	None	None

Clinical presentations	Odynophagia, Dysphagia, Dyspnea	Odynophagia, Localised pain	Odynophagia, Dysphagia, Limit tongue movement	Odynophagia, Dysphagia, Dyspnea	Localized pain, Refer pain to ear	Tongue mass, Localised pain

Duration of symptoms	1 week	1 week	1 week	4 days	1 week	10 days

Prior treatment	None	None	Amoxicillin	None	Amoxicillin	Amoxicillin-clavulanic acid

Physical examination	Marked swelling of BOT, partial occluded OP airway	Antero-lateral tongue swelling & fluctuation	Swelling of FOM & BOT	Swelling of FOM & ventral tongue, partial occluded OP airway	Swelling of BOT & fluctuation with marked tender	Antero-midline tongue mass

Body temperature (°C)	Low grade fever (38°C)	Afebrile (36.6°C)	Afebrile (36.6°C)	Afebrile (37.5°C)	Afebrile (37.3°C)	Afebrile (37°C)

Location of tongue abscess	Rt posterior 1/3	Lt anterior 2/3	Lt FOM & posterior 1/3	Midline FOM & antero-ventral surface	Lt posterior 1/3	Midline anterior 2/3

Deep space of neck infection	None	None	Sublingual abscess	Sublingual cellulitis	None	None

DM: diabetes mellitus, HT: hypertension, FOM: floor of mouth, BOT: base of tongue, OP: oropharynx.

**Table 2:** Investigation, management, and clinical outcome.

	Patient 1	Patient 2	Patient 3	Patient 4	Patient 5	Patient 6
Imaging	CT w/ contrast	None	CT w/ contrast	CT w/ contrast	CT w/ contrast	None

WBC (cell/mm^3^)	9300	4500	14500	12100	5500	5800

Pathogen	*Streptococcus viridans*	No growth	*Acinetobacter lwoffii*	*Beta-haemolytic non-group A,B,D Streptococci spp.*	*Streptococcus viridans*	No growth

Intravenous antibiotics	Amoxicillin-clavulanic acid + ceftriaxone	Amoxicillin-clavulanic acid + ceftriaxone	Clindamycin + ceftriaxone	Clindamycin + ceftriaxone	Amoxicillin-clavulanic acid + ceftriaxone	Amoxicillin-clavulanic acid

Duration for antibiotic (IV + oral form)	2 weeks	10 days	2 weeks	2 weeks	2 weeks	1 week

Anaesthesia	GA	LA	GA	GA	GA	GA

Drainage	Open surgical drainage	Open surgical drainage	Open surgical drainage	Open surgical drainage	Open surgical drainage	Open surgical drainage

Airway management	Tracheostomy	None	ETT	Tracheostomy	ETT	None

Complication	Impending upper airway obstruction	None	Sepsis	Sepsis, Impending upper airway obstruction	None	None

Outcome	Decannulation Day 6 Good	Good	Good	Decannulation Day 5 Good	Good	Good

CT: computer tomographic scan, WBC: white blood count, IV: intravenous, GA: general anaesthesia, LA: local anaesthesia, ETT: endotracheal intubation.

**Table 3:** Summary of the prior case series of lingual abscess.

Year	Authors	No. of cases	Case	Sex	Age (y)	Underlying disease	Source of infection	Clinical presentation (severity)	Abscess location	Sub-lingual infection	Anaesthesia	Drainage
1970	Jain HK, et al	2	No. 1	F	26	No	No	P^*∗*^& S^*∗*^	Middle 1/3	No	LA	Aspiration
			No. 2	F	4	No	No	Pain & S^*∗*^	Anterior 1/3	No	LA	I & D

1996	Jungell P, et al	2	No. 1	M	40	No	No	Pain & S^*∗*^	Middle 1/3	No	LA	I & D
			No. 2	M	51	No	No	Pain & S^*∗*^	Middle 1/3	No	LA	I & D

2004	Antoniades K, et al	3	No. 1	M	55	Thyroid cancer	Trauma	P^*∗*^, S^*∗*^, Dyspnea	Anterior 2/3	Yes	LA	Aspiration+I&D
			No. 2	M	53	Leukemia	Dental	P^*∗*^, S^*∗*^, Dyspnea	Anterior 2/3	Yes	LA	Aspiration+I&D
			No. 3	M	49	DM	Fish bone	P^*∗*^ & S^*∗*^	Anterior 2/3	No	LA	I & D

2004	Balatsouras DG, et al	4	No. 1	F	67	DM	No	Pain & S^*∗*^	Posterior 1/3	No	LA	Aspiration
			No. 2	M	58	No	No	P^*∗*^ & S^*∗*^	Middle +posterior 1/3	No	LA	Aspiration
			No. 3	M	44	No	No	Pain & S^*∗*^	Middle 1/3	No	LA	Aspiration
			No. 4	M	65	DM	No	P^*∗*^ & S^*∗*^	Posterior 1/3	No	LA	Aspiration

2006	Kiroglu AF, et al	2	No. 1	M	7	No	No	P^*∗*^ & S^*∗*^	BOT	No	GA	Aspiration
			No. 2	F	14	No	Fish bone	P^*∗*^ & S^*∗*^	BOT	No	GA	Aspiration

F: female, M: male, P^*∗*^: painful tongue + odynophagia, S^*∗*^: tongue swelling, LA: local anaesthesia, GA: general anaesthesia, I & D: incision & drainage, DM: diabetes mellitus, BOT: base of tongue.

**Table 4:** Clinical manifestation, location of abscess, and management of our case series and case series from literature review.

Variable	Our case series (n=6)	Literature review (n=13)	Total patient number (n=18)
Clinical presentation			
Pain + tongue swelling & no dyspnea	4	11	14
Dyspnea & airway distress	2	2	4

Abscess location			
Anterior 2/3 (+ Middle 1/3)	3	8	10
Posterior (base of tongue)	3	5	8

Sublingual space infection			
Present	2	2	4
Absent	4	11	14

Anaesthesia			
LA	1	11	12
GA	5	2	6

Drainage			
Needle aspiration	-	7	7
I & D	6	4	9
Aspiration + I & D	-	2	2

Tracheostomy			
Required	2	-	2
Non-required	4	13	16

LA: local anaesthesia, GA: general anaesthesia, I & D: incision & drainage.
